# The second complete mitogenome of *Nemipterus virgatus* to dissect control region structure and phylogenetic problem of the superfamily Sparoidea (Teleostei, Perciformes)

**DOI:** 10.1080/23802359.2019.1674708

**Published:** 2019-10-07

**Authors:** Ren-Xie Wu, Yun Zhai, Ben-Ben Miao, Su-Fang Niu, Hao-Ran Zhang, Fang Liu, Chun-Xiao Ou

**Affiliations:** a College of Fisheries, Guangdong Ocean University, Zhanjiang, Guangdong, P. R. China;; b School of Life Sciences, East China Normal University, Shanghai, P. R. China;; c Guangdong Leizhou Rare Marine Life National Nature Reserve, Zhanjiang, Guangdong, P. R. China

**Keywords:** *Nemipterus virgatus*, mitogenome, control region, Sparoidea

## Abstract

In this study, we determined the complete mitogenome of *Nemipterus virgatus* of which the length was 17,073 bp, including 37 canonical mitochondrial genes and 2 non-coding regions. The control region contained termination associated sequence domain (TAS), central conserved domain (CSB-F, CSB-E, CSB-D, CSB-C, and CSB-A), conserved sequence block domain (CSB-1, CSB-2, and CSB-3), and tandem repeat sequence domain (TTD). Nine single nucleotide polymorphisms and three insertion of tandem repeat sequence (each length in 28 bp) were detected between two *N. virgatus* mitogenomes. The phylogenetic analysis showed that the families Nemipteridae, Sparidae, Centracanthidae, and Lethrinidae did not gather into a monophyly of superfamily Sparoidea in the neighbor-joining tree.

The golden threadfin bream *Nemipterus virgatus* (Houttuyn 1782) is one of the most widespread and abundant species of the family Nemipteridae (Teleostei, Perciformes), ranging from southern Japan to northwestern Australia and Arafura Sea (Russell 1990, p. 60). It is a benthic fish that usually inhabits muddy or sandy bottoms of the continental shelf at depths to 200 m, supporting an important commercial marine fishery in the southern part of the East China Sea and the northern South China Sea (Liu et al. 2016. p. 206). The previous studies of *N. virgatus* mainly reported on resource and fishery biology and little is known about its genetic background. Although the complete mitogenome of *N. virgatus* has been sequenced by Wu and Li (2016), its control region structure and the relationships of Nemipteridae and associated families were not analysed. Here we sequenced another complete mitogenome of *N. virgatus* to dissect control region structure and the phylogenetic problem of the superfamily Sparoidea.

One specimen of *N. virgatus* was obtained by longline fishing in October 2010 from Guangdong Leizhou Rare Marine Life National Nature Reserve, Beibu Gulf, the South China Sea (20°38′52″N, 109°43′28″E). It was preserved in 95% ethanol and deposited in Guangdong Ocean University (20101023059). The complete mitogenome of *N. virgatus* (GenBank accession number: KU933270) amplified by designing 15 primer pairs, and it was 17,073 bp in length with a slight bias towards A + T (GC ratio is 42.93%). It contained 13 protein-coding genes, 22 tRNA genes, and 2 rRNA genes, and 2 non-coding regions, with a similar gene order to other nemipterids (Li et al. 2016; Wu and Li 2016; Wu et al. 2016, 2017; Zhai et al. 2019). By comparing with previous *N. virgatus* mitogenome (KR701906; Wu and Li 2016), only 9 single nucleotide polymorphisms (SNPs) were identified, indicating the low level of genetic diversity in the species. Among them, one SNP was detected in each of 12SrRNA, 16S rRNA, *ATPase6* and *ND4* genes, but four SNPs were found in *ND5* gene, suggesting that *ND5* gene has higher variation than other mitochondrial protein-coding genes.

Four domains the termination associated sequence domain (TAS), the central conserved domain (CCD), the conserved sequence block domain (CSB) and tandem repeat sequence domain (TTD), were identified in the control region of *N. virgatus*. According to Guo et al. (2003) report, one extended terminal associated sequence (ETAS: TACATTACTATGTATTATCACCATATTATGTTTTTAACCA), two repeated TAS motif (TACAT) and one complementary TAS (cTAS) motif (ATGTA), were found in the TAS of *N. virgatus*. All the six conserved sequences of CCD, namely, CSB-F, CSB-E, CSB-D, CSB-C, CSB-B, CSB-A, can be identified in *N. virgatus*. The first three are easily identified according to their general sequences described by Lee et al. (1995), while the latter three are recognised according to their core sequences: GCATAAGTT, ATGGCG, CCATGCCGG, respectively. The CSB1 (ATAATTAGAATTCATGAGCATAA) in *N. virgatus* can be determined by its most conserved partial sequence among fishes, while the CSB2 and CSB3 were easily identified by containing two series of C-sequences at TA interval and riching in A and C characteristics, respectively. Nine tandem repeat sequences (TRS), each of 28 bp in length, were detected in the TTD, which were three more than those previously reported by Wu and Li (2016). Such genetic variation supports that the control region could be developed molecular markers for studying the intraspecific micro-evolutionary of *N. virgatus*.

To test the hypothesis that four families Nemipteridae, Sparidae, Centracanthidae, and Lethrinida are classified as a monophyletic group of superfamily Sparoidea (Johnson 1980; Russell 1990), a total of 32 complete mitogenome sequences of the Percoidei were used for constructing bootstrapped neighbor-joining (NJ) phylogenetic tree using MEGA6.06 programme (Tamura et al. 2013). In NJ tree (Figure 1), six species of *Nemipterus* and *Scolopsis* were grouped monophyly of family Nemipteridae at the basal position of Percoidei, while the three associated families (Sparidae, Centracanthidae, Lethrinidae) were gathered into another clade with other 4 families. Obviously, this study does not support the hypothesis that the four families should be classified as a superfamily Sparoidea, although they have morphological similarities.

**Figure 1.**
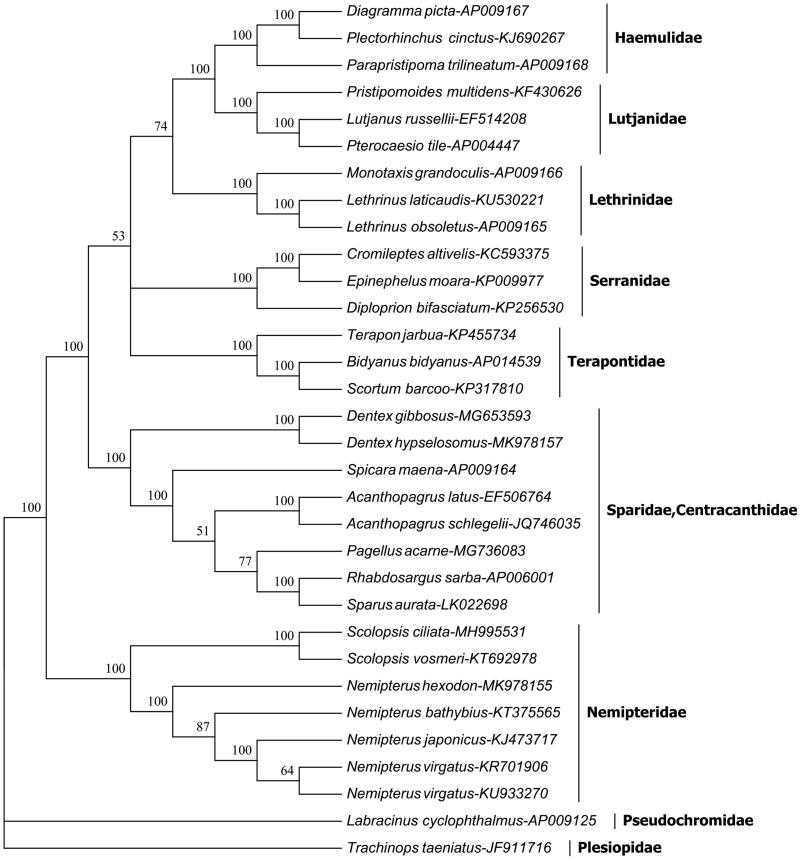
The neighbor-joining phylogenetic tree of *Nemipterus virgatus* and other 30 species of the Percoidei based on their complete mitogenome sequences. The bootstrap value was given for each branch.
